# Life and Living

**Published:** 1889-11-09

**Authors:** 


### The Doctor's Pulpit.

### LIFE AND LIVING.

We have here to deal with man's physical condition rather than with his spiritual life. Yet the doctor cannot ignore the latter, for it constitutes by far the higher and better part of our earthly life, whilst it pervades the whole existence of every member of the human race. Martial has well said, "Life is not to live, but to be well." Well, that is in heart and soul as well as in body. There can be no sound bodily health in the highest sense unless the spiritual man is also alive and well. The feeling of God always, everywhere, and in all, if ever present in a man's thoughts, will secure to him a strength and happiness which nothing else can give him. The *mens sana in corpore sano* includes not only attention to bodily health, but the fullest consciousness that life is no idle dream but a solemn reality. As Carlyle says, "Life is thy own; it is all thou hast to front eternity with." And life, like every other blessing, derives its value from its use alone. Thus the doctor may specially direct attention to the proper uses of life, knowing how sadly they are forgotten in our day and generation. Further, we ought to realise that to live long is to live slowly; not to be too much addicted to one thing, but to husband our strength, develop our reasoning faculties, improve our knowledge, extend our influence for good, cultivate habits of truth, justice, industry, and simplicity, and strive by example, and by unhasting, yet unresting work, to leave the world a little better than we found it. He who does this will surely find health and happiness throughout his life, be it long or short. Indeed, to strive to live so is to become indifferent to the risks, trials, and vexations we must all encounter, because the whole system of such a man, by God's mercy, will be sound, and neither fear of death nor hope of reward will cause him to alter his course. Life properly regarded, as Cicero long ago pointed out, is, after all, but a loan. Men on earth are at best but children at school, and the knowledge they acquire of the losses accruing to them from neglected opportunities in boyhood and youth, should at least cause them to husband their lives to the utmost, in view of the great hereafter, for which we all should be in a state of preparation—each responsible for himself and each master of his own actions. Well would it be if men and women in this nineteenth century had a better, a more personal and real sense of these elementary facts of our existence, for then they would surely cease to exist only, as so many of them do, on the theory that they must make haste to eat and drink for to-morrow we die.

As physicians, we have to study the whole duty of man, not this portion or that, but the whole of man's nature. Long practice makes us able very often to diagnose the disease of a particular patient by observation alone, without examination, and before a single question has been asked. This is literally true of man's physical condition, and it is equally true of his spiritual condition too. For as the trained physician can often tell at a glance what his patient's ailment is, so can an experienced missioner discern in the features of the penitent who consults him what vicious habit or special weakness he has given way to. This may seem strange to many readers, but it is precisely true in fact, and conveys a moral we need not further enforce. Free will entails that men and women may become the victims of their own vices, if they have neither the courage nor the desire to fight manfully against them. Let us take a few notorious instances of this, by way of illustration, from every-day life.

Take the man of business. Does he meddle with no man's business but his own? Work moderately, live regularly and take none but innocent pleasures freely? Not a bit of it. On the contrary, how many men in the race to be speedily rich debase themselves by touting to those who they believe able to give them business, work not only immoderately but beyond their strength, neglect all regular habits of feeding and exercise, and take their pleasures during the hours they ought to devote to necessary sleep. No wonder if the harassed system and worried brain makes such men seek relief in continual "nipping" or immoderate smoking. Always on the drive, they forget everything except the golden apple, which, when it is grasped, may, and too often does, prove to be very dust and ashes. What would our fathers and grandfathers think if they could see their sons as the doctor sees them, and what must be the effect upon wives and families? Business from morning to night, with the exception of a few brief days, now and again, which cannot bring either enjoyment or rest, in many cases, because the physical strain has been so great as to destroy, or at any rate to suspend, the sense of enjoyment or repose. Even Sunday with such men is either taken up with thoughts of business and its actual performance, or spent in a houseful of people where tennis, boating, card playing, and billiards engage the company from morning till late at night. What can the doctor do for such men as these? He must either stop their mad career or the patients will be stopped for ever. Yet the more madly such a man is rushing to his doom the more is he impressed with the feeling that he cannot leave his business or alter his course for a moment, or he will be ruined. A famous physician once had such a patient to deal with. He advised a sea voyage, and cessation from all business for six months. His patient said it was impossible to follow such advice, could he do nothing else? Yes, said the physician. You can take a beefsteak at one o'clock each day, with a pint and a half of stout. Do this for a fortnight, and come and see me at the end of it. The patient returned, and begged to be allowed to discontinue the steak and stout, as they made him so sleepy; but the physician was obdurate, and kept up the treatment for six weeks, by which time his patient felt he could not do any business of importance after lunch, and so consented to go for the sea voyage. He returned sound in body and mind, and no one could be more sorry, than the patient, for the insane folly of his former mode of life, which he has lived to repent and avoid. Would that all others similarly situated—and their number is largely on the increase—could be similarly clothed and brought to their right mind.

Bad as is the condition of such men of business as those we have just described, that of some of the women of fashion of to-day who are leaders in their own circles is infinitely worse. The introduction of American women into English society has not been an unmixed blessing. This was admitted, quite recently, by an American gentleman of knowledge and position, who said he had no doubt that the freedom allowed to American women in their own country, where custom had created many unwritten but necessary safeguards, had set a new fashion in London society, where no such safeguards exist, which had now degenerated into licence. Not only is the ambition of certain well-known leaders in society to secure a husband of position and influence, but to have afterwards in habitual attendance a body of gentlemen, one of whom they select for their special favours, and denominate their "best young man." Husbands tolerate this practice, and some even encourage it; the ladies themselves take entire possession of the "best young man," and have been known to call upon his *fiancée* and boldly declare that the engagement must be given up, as they would not tolerate any rival to themselves, though married women. Who can wonder at the scandals resulting from such a system, or the premature breaks down in health which frequently result to the women who live only for the hour, and waste their beauty, talents, health, character, and life in living not only without God, but without hope even in this world. Smoking, a glass of wine or brandy at any and every hour without consideration or hesitation, conversations which demoralise and degrade, neglect of home duties and of every serious purpose in life, such is the condition of the leaders of certain fashionable circles, who are unfortunately increasing rather than diminishing in numbers at the present time.

Take again the position of the millionaires of to-day and that also of many very wealthy men and women besides. What an immensity of good they could do in what remains to them of life if they would only rouse themselves and act. What a trouble the money is to some of them, and how fearfnl they are lest they should lose it. The lives some of these people live are notorious, and no real man or woman can envy, because they are forced to pity them, in their self-guarded miseries. Would that they could be brought to know we live in deeds, not years, in thoughts, not breaths. He most lives, as Bailey has it, who thinks most, feels the noblest, acts the best.

We could wish that every reader, and especially every youthful reader, would ponder these things, take a resolution, and adhere to it throughout life. How much vexation they would be saved if they would but remember when starting upon life's journey—

In this the art of living lies,
To want no more than may suffice,
And make that little do.

No one who strives to live for himself alone can be said to live at all. He only can truly be said to live who is engaged in some laudable pursuit, and who acquires an honourable name by his actions, his art, or nobility of character. There is an old but golden rule of healthy physical life. Take exercise to keep your feet warm, be temperate to keep your brain cool, eat only when you are hungry, and drink but enough to satisfy nature's requirements. This done, forget not that you are a responsible being, with responsible duties and all that they entail. "Work, therefore," as Carlyle has it, "even as he has done, and does, like a star, unhasting, yet unresting. Thy life, wert thou the pitifullest of all the sons of earth, is no idle dream, but a solemn reality."

